# Analysis of Plant Diversity and Importance Value Index in Central Ethiopian Agroforestry Systems

**DOI:** 10.1155/sci5/9959255

**Published:** 2026-04-15

**Authors:** Admasu Moges

**Affiliations:** ^1^ Department of Biology, College of Natural and Computational Sciences, Debre Berhan University, P.O. Box 445, Debre Berhan, Ethiopia, dbu.edu.et

**Keywords:** diversity indices, home garden, native species, parkland, vegetation structural analysis

## Abstract

Despite offering multiple benefits, agroforestry practices have declined due to uncertain landownership, population growth, and limited awareness and scientific data. This study aimed to investigate the composition, diversity, and importance value index (IVI) of woody plant species in home gardens (HGs) and parklands in three districts of the North Shewa Zone. The study used surveys and sampling techniques to collect data from 80 HGs to 33 parklands. Species richness, Shannon diversity, and evenness were analyzed using R software with Version 4.2.2. Their mean values were compared between the two agroforestry systems and among districts using Tukey’s pairwise comparison in SPSS (Version 20) statistics at a *p* value < 0.05. Additionally, the IVI was used for the structural analysis of woody species. The findings showed that 136 plant species from 58 families were identified. The Fabaceae family dominated in both HG and parkland practices. Trees were the most common growth form. HGs showed higher diversity (114 species) compared to parklands (79 species). Species richness also varied by districts, with Tarmaber having the most (86) and Ensaro the least (42) for HGs, and Tarmaber (37) had more than Mojana‐Werena (21) for parklands. Tarmaber also scored the highest Shannon diversity for HGs (1.94) and parklands (1.74), followed by Mojana‐Wedera. Overall, HGs displayed significantly higher richness and diversity than parklands across the study area at a *p* < 0.05. Interestingly, parkland data showed no significant differences in diversity metrics. The study also revealed 61.03% native (indigenous and endemic) and 38.24% exotic species. This growing interest in including exotic species necessitates addressing potential ecological disruptions while promoting overall plant diversity. The first five most frequent species in the sampled HGs were *Rhamnus prinoides*, *Croton macrostachyus*, *Eucalyptus globulus*, *Schinus molle*, and *Ziziphus spina-christi*, which occurred in 20.18%, 7.89%, 5.26%, 5.26%, and 4.39% of all HGs, respectively. Similarly, the most frequent species in parklands were *C. macrostachyus*, *E. globulus*, *Olea europaea*, *Erythrina brucei*, and *Juniperus procera*, with occurrence rates of 27.85%, 12.66%, 11.39%, 10.13%, and 10.13% of all sampled parkland, respectively. Based on the IVI, *R. prinoides* (265.11%) emerged as the dominant in HGs, followed by *E*. *globulus* (85.53%), *Cupressus lusitanica* (17.27%), *C*. *macrostachyus* (13.45%), and *Z*. *spina-christi* (9.53%). In parklands, *E. globulus* (142.32%) had the highest IVI, followed by *C*. *macrostachyus* (33.53%), *A*. *abyssinica* (18.13%), *Z. spina-christi* (17.46%), and *O*. *europaea* (14.28%). These findings highlight the ecological significance of these species in their respective habitats. Finally, the identification of plant species in both HGs and parklands provides valuable information for conservation efforts and the development of effective land management practices.

## 1. Introduction

Agroforestry is a long‐established and highly adaptable land‐use system that integrates trees or shrubs with crops, livestock, or both on the same land [1–3]. Rooted in indigenous knowledge [4], it is increasingly recognized for its contributions to soil fertility improvement [5], biodiversity conservation [6]), and enhanced rural livelihoods. Intensive agroforestry systems deliver multiple ecological and socioeconomic benefits, including improved soil health, biodiversity conservation, and carbon sequestration ranging from 12 to 228 Mg ha^−1^ [2, 7]. They also provide a wide range of products—such as timber, fuel wood, poles, medicinal resources, and food—thereby strengthening household resilience. Globally, agroforestry systems take diverse forms, including parklands (forest farming), home gardens (HGs), windbreaks, alley cropping, silvopasture, riparian buffers, mixed intercropping, and crop‐specific systems such as coffee‐, enset‐, and fruit‐based agroforestry [2, 8–10]. In tropical Africa, HGs, mixed intercropping, and integrated tree–pasture systems are particularly common, notably in Kenya [11] and Senegal [7].

In Ethiopia, agroforestry is widely practiced through systems such as parklands, HGs, farm boundary plantings, and woodlots [12–14]. Among these, HGs and parklands are the most prevalent, while many woodlots are dominated by eucalyptus monocultures. Consequently, HGs and parkland systems play a central role in conserving biodiversity and supporting local livelihoods [14–16]. HGs, typically established around homesteads and characterized by high species diversity, differ from parklands, which feature scattered trees within cultivated fields [17–19]. Overall, these systems enhance ecosystem services by increasing biodiversity, diversifying income sources, and improving soil and water quality through reduced erosion and flooding, ultimately contributing to more sustainable and resilient livelihoods for Ethiopian farming households [2].

Despite its fundamental benefits, agroforestry remains poorly adopted by many Ethiopian farmers, and some practices are even declining [2, 20]. This limited application is driven by multiple, interrelated constraints, including insecure land tenure and unclear land boundaries that discourage long‐term investment in tree planting [21], limited water access, low education levels, inadequate training and technical knowledge, poor access to seedlings, markets, and extension services, low seedling survival rates [2, 3, 22], and shortages of skilled and unskilled labor. These challenges are further aggravated by the continued emphasis of extension services on monoculture systems, with agroforestry receiving little attention, leaving small‐scale farmers with limited confidence to adopt new practices [23]. At the same time, the diversity and structure of agroforestry systems are shaped by agroecological and socioeconomic factors, yet research on these aspects remains limited and is largely confined to district‐level studies or university theses, which fail to capture broader zonal or regional patterns [19, 20, 24]. Consistent with this, species diversity varies across agroforestry systems and regions in Ethiopia [6], and the lack of research evidence and community awareness has contributed to tree removal from parklands, HGs, and grazing lands during social interventions, particularly in the North Shewa Zone (NSZ). Moreover, extensive small‐scale farming and expanding human settlements in the central Shewa plateau have accelerated landscape fragmentation and land degradation [14].

To address the challenges facing agroforestry in Ethiopia, interventions are needed to enhance extension services, improve access to indigenous tree seedlings, and stimulate the development of higher‐value tree products [25]. Additionally, scientific information is crucial for bridging knowledge gaps and promoting sustainable agroforestry practices. However, the conservation of plant diversity within agroforestry systems has been largely overlooked [6]. To fill this knowledge gap, conducting botanical studies to analyze the diversity, composition, and structure of woody plant species in agroforestry systems, such as HGs, is essential [26]. This information is vital for maintaining biodiversity, increasing agroecological resilience, and providing local communities with essential agroforestry products, especially during times of food scarcity and drought. Thus, the overall objective of this study was to evaluate the contribution of HGs and parklands to biodiversity conservation in the three districts of NSZ. The specific objectives were (1) to identify the plant species and evaluate their composition and diversity in the two agroforestry systems across three districts, (2) to compare the plant diversity indices’ values between the two agroforestry systems as well as among three districts using multivariate analysis, and (3) to analyze the importance value index (IVI) of the woody species in HGs and parklands of study area to estimate ecologically and economically dominant and important plant species.

## 2. Materials and Methods

### 2.1. Description of the Study Area

**FIGURE 1:**
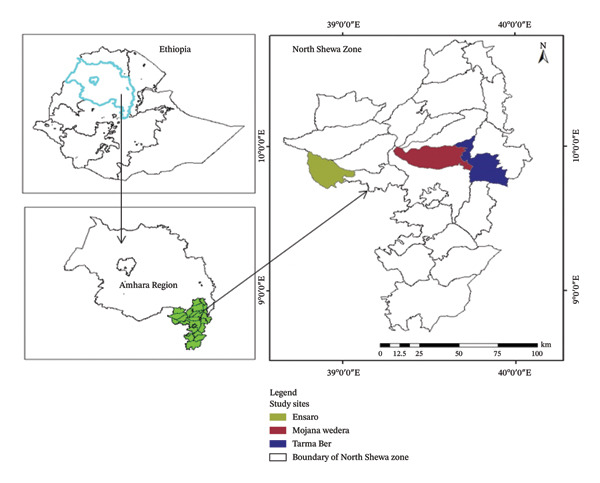
A map of the study area in North Shewa Zone, Amhara Region, Ethiopia.

The study area is located in NSZ, one of the 12 zones within the Amhara National Regional State of Ethiopia. The zone’s capital city, Debre Birhan, lies 130 km northeast of Addis Ababa, the national capital. Geographically, NSZ is situated between 8° 38′ and 10°42′ north latitude and 38°40′ to 40°03′ east longitude. The zone encompasses 32 districts (according to the 2020 report of the NSZ Department of Communication Affairs). Three districts—Mojana‐Wedera, Tarmaber, and Ensaro—were chosen for the study (see Figure 1). These districts are located 180, 190, and 130 km from Addis Ababa, respectively, and 50, 60, and 85 km from Debre Birhan town. The altitude range within these districts varies from 1100 to 2887 m above sea level. The study area resides in the central highlands of Ethiopia, bordering the western escarpment of the Great Rift Valley, which bisects the country. This location, with its diverse soils, climate, and topography, influences the variety of vegetation and agricultural practices found in the region. The zone borders the Oromia Region to the south and to the west, the South Wollo Zone to the north, the Oromo Nationality Special Zone to the northeast, and the Afar Region to the east. Agriculture is the primary economic activity and source of livelihood for the zone’s residents. Thus, farming and cattle rearing, often practiced together as mixed farming, are the most common agricultural practices in the selected districts. Furthermore, these districts experience maximum and minimum rainfall ranging from 650 to 1870 mm, with temperatures varying between 10°C and 31°C.

### 2.2. Study Design

This research employed vegetation and descriptive surveys as they are the most suitable methods for data collection. Accordingly, a systematic sampling design [27, 28] was used to gather vegetation data, including plant specimens, in both parkland and HG agroforestry systems. Since the analysis included both numerical (quantitative) and descriptive (qualitative) data, the researcher used a combination of quantitative and qualitative approaches.

### 2.3. Site Selection

This study was conducted in three districts of the NSZ, Amhara National Regional State, Ethiopia, namely, Mojana‐Wedera, Tarmaber, and Ensaro (Figure 1). These districts were purposefully selected due to their variation in agro‐ecological zones and the prevalence of both HG and parkland agroforestry practices. Information on the number of rural kebeles (kebele is the smallest administrative unit) in each district was obtained from the perspective district websites and through communication with experts from each district’s office and Zone’s Department of Communication Affairs. Accordingly, Ensaro, Mojana‐Wedera, and Tarmaber, districts have 13, 14, and 23 rural kebeles, respectively. Based on the extensive presence of agroforestry practices, particularly HGs and parklands, two kebeles were chosen from each of Ensaro and Mojana‐Wedera and four were chosen from Tarmaber. A total of 80 HGs were then randomly selected, with 10 households participating from each chosen Kebele. Parkland, however, is not uniformly distributed across farmlands. Therefore, 45‐ha parklands were purposefully selected across eight kebeles. Generally, 80 HGs and 45‐ha parklands were selected using a multistage sapling techniques.

### 2.4. Sampling Design and Field‐Based Plant Inventory

In order to collect vegetation data from HGs and parkland (crop lands), different sampling designs were employed. For a HG agroforestry system, a complete enumeration of the plant species was made [18, 29] since the size of the HGs of an individual farmer is relatively small. Hence, for determining the plant species diversity of the HGs, 80 randomly selected households’ HGs (10 HGs x 8 Kebele) were assessed. However, to collect vegetation data from parkland and its adjacent, transect‐quadrat sampling methods using systematic sampling design were applied. Accordingly, four sampling plots with 50 m × 100 m sample size quadrates [17, 20, 29] were systematically laid out along transects, which are crossing the adjacent individual parklands. Large area size sample plots for parklands were used since it is less likely to get woody species from small plots of the parkland system [18]. Accordingly, a total of 32 rectangular quadrats (4 quadrats × 8 Kebele) of 45 ha size were laid out. The distance between two consecutive sampling plots along a transect line within the parkland was 100 m. In each HG and quadrat of parkland, all plant species encountered were counted, sampled, and identified. To calculate the basal area (BA) of woody plants in each quadrat, the diameter at breast height (DBH) was measured for all trees and shrubs. In this study, trees and shrubs were defined as woody plants with a DBH of at least 2.5 cm and a height of at least 2 m. Specifically, a tree was characterized as a woody perennial plant with a single main stem and has more or less a definite crown. In contrast, shrubs were identified as woody perennial plants that often lack a distinct crown and have several stems growing from the same root. Moreover, saplings, defined as trees with diameters less than 2 cm and heights at least 1 m, were measured. Similarly, seedlings, with heights less than 1 m, were also included in the data collection. Height measurements were taken using a combination of measuring tape and ocular estimation. Additionally, the aerial ground cover‐abundance value of each plant species was determined in percent (%) using ocular estimation and immediately converted into the Braun‐Blanquet (BB) scales ranging from 1 to 9 (as modified by van der Maarel [30]: 1 for one to three individuals; 2 for few individuals (0.5–1.5%]; 3, (1.5–3%]; 4, (3–5%]; 5, (5–12.5%]; 6, (12.5–25%]; 7, (25–50%]; 8, (50–75%]; 9, (75–100%].

Additionally, the plant specimens were collected from selected agroforestry practices (HGs and parklands) and then recorded using their vernacular names being assisted by land owners. Then, the specimens were numbered, pressed, dried, and taken to DBU for identification. Identification of specimens was carried out by using the Flora of Ethiopia and Eritrea [31–40]. Further identification was also made in Herbarium of Addis Ababa University (ETH) with the help of an expert and authenticated specimens. Finally, the voucher specimens were deposited at the University.

### 2.5. Data Analysis

#### 2.5.1. Descriptive Statistics

Descriptive statistics such as percentage and frequency were employed to describe and summarize the results using figures and tables via applying Microsoft Excel spreadsheet 2010 and Statistical Package for the Social Sciences (SPSS) with Version 20.

#### 2.5.2. Plant Diversity Indices’ Analysis

The diversity and structure of plant species were analyzed via quantitative analysis. To determine the species richness, Shannon diversity index, and evenness, the R statistical software with Version 4.2.2 was applied. Accordingly, species richness is a biologically appropriate measure of alpha (α) diversity and is usually expressed as a number of species per sample unit [41]. The Shannon diversity (*H* ^′^) and evenness (E′) indices are calculated as a measure to incorporate both species richness and species evenness [28, 42]. The Shannon diversity index (*H* ^′^) was therefore calculated from the following equation:

(1) H′=−∑i=1spilnpi,

where *H* ^′^ = the Shannon diversity index, Pi = the abundance of the *i*th species expressed as a proportion of total cover, ln = the log base “*n*,” and S = the number of species in the community [28, 42]. The values of the Shannon diversity index are usually found to fall between 1.5 and 3.5 and only rarely surpass 4.5 [28, 42].

Pielou’s evenness index (*J*) [43] was calculated from the ratio of observed diversity to maximum diversity using the following equation:

(2) J=H′H′max=H′lnS,

where *J* = Pielou’s evenness index, *H* ^′^ = the Shannon diversity index, *H* ^′^max = the maximum level of diversity possible within a given population, which equals ln*S* (where “*S*” is the number of species). Pielou’s evenness index (*J*) is normal between 0 and 1, with 1 representing a situation in which all species are equally abundant.

Sorenson similarity coefficient (SSC) index is preferred to that of the Jaccard as it gives more weight to the species that are common to both sites than to the unique species to either of the communities. This index ranges from 0 (No Similarity) to 1 (Complete Similarity) corresponding to 0–100% [44].

(3) SSC=2a2a+b+c,

where SSC = the Sorenson similarity coefficient, *a* = the number of species common to both sampling sites (i.e., the two surveyed agroforestry sites), *b* = the number of species in one of the sites to be compared (in the present study case, it is the HG), and *c* = the number of species present in the other community (that is the parkland).

#### 2.5.3. Comparing Diversity Indices Using Parametric Tests

To compare the Shannon diversity indices [richness (taxa‐s), Shannon diversity (Shannon‐H′), and evenness] across HGs and parkland plots in all three districts, a two‐way multivariate analysis of variance (MANOVA) was employed. This parametric test, based on a general linear model, was chosen because data for all Shannon diversity indices exhibited normal distributions in both HG and parkland plots, as visualized in simple scatter plots (Figure A1). However, the MANOVA provided information on the overall variation in the Shannon diversity indices across the entire study area. Therefore, to determine significant differences between any two of the three districts in terms of specific Shannon diversity indices (taxa‐S, Shannon‐H′, or evenness), pairwise comparisons using post hoc tests were conducted using SPSS with Version 20.

#### 2.5.4. IVI for Woody Plant Structural Analysis

The IVI percentage (IVI%) provides a valuable tool for evaluating the relative significance of individual tree species within agroforestry systems, aiding in understanding their structural contributions [45]. For calculating this one, the percentages of relative frequency (RF), relative density (RD), and relative BA (RBA) were applied using Kent [28] procedures, as presented below. However, to calculate the percentages of these relative index values, the parameters such as height, frequency, BA, and density must be analyzed using the following formulas. Thus, for quantifying the density, BA, and frequency, thereby IVI for HGs, the average sample size of the whole study area and across the three districts was calculated (Table 1). Frequency, density, and BA of woody species were calculated following procedures of Curtis and Mcintosh [46].

(4) Frequency=no. of quadrats in which a species occurs total no. of quadrats examined,

(5) RF=the number of plots where a species occur the total occurrence of all species in all of the plots used during the study×100,

(6) density=total no. of individuals of a species foundtotal area examined,

(7) RD=the number of all individuals of a species the number of all individuals of all species×100,

(8) BA=πD24, where π=3.14; d=DBH m,

(9) RBA=the basal area of a species Total basal area×100,

(10) IVI=RF+RBA+RD.

**TABLE 1:** The number of sampled Kebeles, home garden number (HG no.), parkland, and their area size and altitudinal range in meters (m).

Variables	Studied districts		
	Ensaro	Mojana‐Wedera	Tarmaber
Sampled kebeles	2	2	4
Surveyed home garden (HG) no.	20	20	40
Area size range of HG (m^2^)	1250–5000	1200–5000	100–5000
Average sampled size of HG (m^2^)	2563 m^2^	3100 m^2^	1100 m^2^
Altitudinal range of HG (m)	1331–2269	2393–2664	2038–2830
Area size of surveyed parkland (m^2^)	350,000	350,000	350,000
Sampled size of parkland (m^2^)	1 (50 × 100) × 4 × 2 = 8	1 × 4 × 2 = 8	1 × 4 × 4 = 16
Altitudinal range of parkland (m)	1354–2127	2387–2662	2038–2830

## 3. Results and Discussion

### 3.1. General Features of HGs and Parkland

The area size of the HGs ranged from 100 to 5000 m^2^ with an altitudinal range of 1331–2830 m. Of course, the sample size of surveyed parkland was already fixed (Table 1). The parkland was also located at an altitudinal range of 1354–2830 m.

### 3.2. Area Size Categories of Agroforestry Practices Across the Three Districts

The sampled HGs were classified into three categories: small, medium, and large, based on their relative area size (Table 2). The majority (60%) of HGs were small, ranging from 0.01 to 0.24 ha (ha). Conversely, large HGs (≥ 5000 m^2^) were the least common, accounting for only 17.5% of the sample. This implies that the majority of the households of the study area had small size HGs, which might be due to the scarcity of land and population increase, despite variations among districts. More specifically, the average area of HGs in Ensaro, Mojana‐Worena, and Tarmaber districts was 2563 m^2^, 3100 m^2^, and 1100 m^2^, respectively. This indicates that the average HG size in Tarmaber District was relatively the smallest, compared to the other districts. This could be attributed to the higher population density in the chosen sites (kebeles), which were situated around the urban center of Debre Sina, the capital of Tarmaber District, while the opposite was true for Mojana‐Worena District. Overall, the total area of all HGs in the study area was 157,780 m^2^ (15.78 ha), with an average area of 1972 m^2^ (∼0.2 ha) per HG. Mengistu and Fitamo [47] reported a different range for HG size in Dilla Zuria District, southern Ethiopia, with an average of 665.42 m^2^ and a range of 250 m^2^–2000 m^2^. This suggests that the HGs in this study area were larger than those in Dilla Zuria District. This difference might be due to the relatively high population density in southern Ethiopia, leading to smaller landholdings for HGs.

**TABLE 2:** The size of home gardens (HGs) across the three districts of the study area.

HG category	HG area size range	Number of HGs			Total in	
		Ensaro	Mojana	Tarmaber	Number	Percentage
Small	0.01–0.24 ha	10	6	32	48	60
Medium	0.25–0.49 ha	6	6	6	18	22.5
Large	≥ 0.50 ha	4	8	2	14	17.5
Total (average)	15.78 ha (0.197 ha)	20	20	40	80	100

Unlike HGs, the parkland area size was fixed for the study. The survey was conducted in a total sample area of 16 ha across eight kebeles, with each Kebele having a designated area of 0.5 ha (Table 1).

### 3.3. Plant Composition in the Study Area

A variety of plant species were found growing in both HGs and parklands within the studied area of the NSZ in central Ethiopia. A total of 114 and 79 plant species were identified from sampling sites of HGs and parklands, respectively, across three selected districts within the NSZ. This resulted in a total of 136 plant species documented in the study area, belonging to 108 genera and 58 families (Tables 3 and A1). Among the 58 plant families found in this study area, Fabaceae was the most prevalent, comprising over 15.44% (21 species) of the total identified species (Table 3). Poaceae (9.56%) and Rutaceae (4.46%) followed as the next most dominant families. The remaining families were represented in percentages ranging from 3.68% to 1.47% (Table 3). Previous studies by Legesse and Negash [48], Molla et al. [22]), Beyene et al. [20], and Guzo et al. [14], also identified Fabaceae as the dominant plant family in agroforestry systems of Kachabira, Wondo, and Tembaro special districts in southern and Midakegn District in central Ethiopia, respectively. Similarly, Badji [7] reported Fabaceae as the predominant family in HGs in Senegal. The prevalence of Fabaceae plants in the region could be due to a combination of factors, including the suitability of the local climate and soil for their growth, as well as the farmers’ desire to cultivate these plants.

**TABLE 3:** List of families with their corresponding number, and percentage of genera and species.

Family	Plant genera		Plant species	
	Number	Percentage	Number	Percentage
Fabaceae	11	10.19	21	15.44
Poaceae	11	10.19	13	9.56
Rosaceae	5	4.63	5	3.68
Solanaceae	5	4.63	5	3.68
Lamiaceae	3	2.78	5	3.68
Anacardiaceae	3	2.78	4	2.94
Asteraceae	3	2.78	3	2.21
Euphorbiaceae	3	2.78	3	2.21
Malvaceae	3	2.78	3	2.21
Meliaceae	3	2.78	3	2.21
Apocynaceae	2	1.85	2	1.47
Brassicaceae	2	1.85	2	1.47
Celastraceae	2	1.85	2	1.47
Cucurbitaceae	2	1.85	2	1.47
Cupressaceae	2	1.85	2	1.47
Moraceae	2	1.85	3	2.21
Musaceae	2	1.85	2	1.47
Myrtaceae	2	1.85	4	2.94
Rhamnaceae	2	1.85	2	1.47
Rutaceae	2	1.85	6	4.41
Cyperaceae	1	0.93	3	2.21
Alliaceae	1	0.93	2	1.47
Polygonaceae	1	0.93	2	1.47
Sapindaceae	1	0.93	2	1.47
Tiliaceae	1	0.93	2	1.47
Others (each with one species)	33	30.56	33	24.26
Total	108	100.02	136	100.00

When examining the dominant plant families in HGs and parklands separately, of the 58 total families in the study area, 16 and 5 families were exclusive/unique to HGs and parklands, respectively. The remaining 37 families were found in both habitats. However, Fabaceae (with 16 species) was the most prevalent family in HGs, comprising 30.19% of the total plant diversity. In parklands, Fabaceae (with 14 species) was also the dominant family, representing 33.33% of the total diversity. Anacardiaceae and Poaceae each accounted for 8.70% of parkland flora, while the remaining families made up minor portions (4.35% each). Legesse and Negash [48] similarly reported Fabaceae as the most dominant family in the HGs, parklands, and live fences of the Kachabira District in southern Ethiopia. Similarly, when examining the species composition in the present study, 57 of the 136 total plant species (over 42%) were unique to HG areas, while only 22 species (16%) were found exclusively in parklands. This indicates that HGs in the study area possess a higher diversity of plant species compared to parklands. The greater species richness in HGs is likely attributed to the multiple uses these gardens serve for local communities, as noted by Mengistu and Fitamo [47]. The remaining 57 species (42%) were shared by both HGs and parklands.

These consistent findings suggest a preference among local communities in the present study area to remain and cultivate leguminous plants within their HGs and parklands, alongside economically important species like *Rhamnus prinoides* and *Eucalyptus* species (Figure A2). This preference for Fabaceae among farmers might be due to their inherent resilience to disturbances and their adaptability to local environmental conditions, as suggested by Beyene et al. [20], alongside their crucial role in boosting soil fertility.

Still, when comparing the present study to previous ones mainly conducted in southern Ethiopia by Mengistu and Fitamo [47], Legesse and Negash [48], and Tesfay et al. [6], which reported 75, 59, and 52 plant species, respectively, in Dilla Zuria District, Kachabira District, and southeastern rift–valley landscapes of Gedeo Zone (more specifically in the Dilla Zuria District), the present study found a higher species richness (136). Similarly, Beyene et al. [19] documented 22 plant species in the Bluen District of northwestern Ethiopia. More recently, Molla et al. [22] have identified 90 woody species in the Wondo District of southern Ethiopia. These findings suggest a greater diversity of plant species in the central Ethiopian region compared to the northern and southern parts. This difference could be explained by variations in the size of the HGs studied. The HGs in the present study were generally larger (Table 1) than those in the southern and northern regions, and the inclusion of multiple districts may also have contributed to the higher species richness.

### 3.4. Plant Growth Forms in the Study Area

**FIGURE 2:**
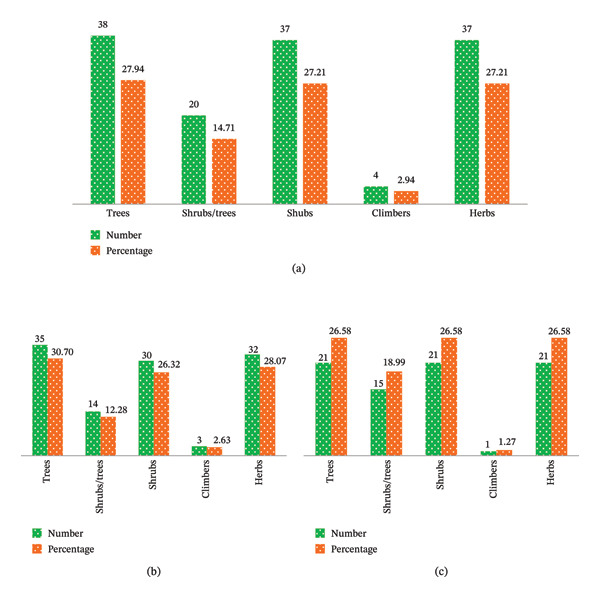
Growth forms of the species found across the whole study area (a) and in both HGs (b) and parkland (c). (a) Growth form in both HGs and parklands. (b) Growth form in HGs. (c) Growth form in parklands.

Based on the distribution of growth forms for plant species, trees were the most dominant growth form (27.94%), followed by shrubs and herbs (27.21%, each) (Figure 2(a)). Contrarily, the least prevalent growth form in the study area was climbers (2.94%). Similarly, the most dominant plant species growing in HGs were trees (30.70%), followed by herbs (28.07%) and shrubs (26.32%) (Figure 2(b)). Climbers were the least common growth form (2.62%). These findings are consistent with those of Mangistu and Fitamo [46] and Legesse and Negash [48], who also reported that trees were the dominant growth form in southern Ethiopia, followed by shrubs, herbs, and climbers. This dominance of woody plants, particularly trees and shrubs, may be explained by their economic importance. Species like *Eucalyptus*, *Citrus*, and *R. prinoides* provided valuable income sources as fuel wood, food/beverages, and construction materials. Moreover, farmers in the study area might be obliged to cultivate these woody species in their HGs due to limited access, scarcity, and high costs of these resources from other forests and markets. However, many of the herbaceous plants grown in HGs were vegetables, including *Ruta chalepensis* L. (Tenadam in their local Amharic name)*, Ensete ventricosum* (Koba), *Capsicum annum* L. (Berberie), and *Allium cepa* L. (Qey shinkurt).

Likewise, the growth forms of the plant species growing in parkland had almost similar patterns to that of HGs. The trees, shrubs, and herbs were equally dominant (26.58%, each) (Figure 2(c)). Contrarily, climbers were the least abundant growth form. The majority of the trees and shrubs in the parklands belonged to the Fabaceae family, including *Vachellia abyssinica* Hochst. Ex Benth, *V. seyal* Del., *V. albida* Del., and *Erythrina brucei* Schweinf. Herbs were primarily monoagricultural crops like *Eragrostis tef* (Zucc.) Trotter, and *Zea mays* L., along with some grasses like *Cenchrus purpureus* (Schumach.) Morrone and *C. geniculatus* Thunb. in the boundary or along the bunds of parkland (Figure 2(c)). This trend of growth form distribution and Fabaceae dominance aligns with the findings of Beyene et al. [20] from Tembaro District of the southern Ethiopia.

### 3.5. Origin of the Plant Species Growing in the Study Area

A comparative analysis in terms of the origin of plant species diversity in HG and parkland agroforestry systems revealed a predominance of native [indigenous plus endemic species (61.03%)], followed by exotic species (38.24%) (Figure 3(a)). Notably, 29 indigenous species were exclusively found in HG systems, while 18 were unique to parkland agroforestry (Figure 3(b)). However, 30 indigenous species were observed in both habitats, indicating shared plant diversity. Among exotic species, 27 were identified solely in HG systems, and 3 in parkland agroforestry, with 22 species being present in both (Figure 3(b)). As also reported by Guzo et al. [14] and Tesfay et al. [6], many of the species identified from the Midakegn District of the Western Shewa Zone (87%) and in southeastern rift–valley agricultural landscapes of Gedeo Zone (63.5%) were native woody species. Furthermore, this study identified six endemic plant species to Ethiopia, including *E*. *tef*, *Laggera tomentosa* (Sch. Bip. ex A. Rich.) Oliv. & Hiern, *Impatiens rothii* Hook. F., *Millettia ferruginea* (Hochst.) Bak., *Rumex nervosus* Vahl, and *E*. *brucei*. These six endemic species represented 4.41% of the total identified species and were higher than the other endemic species identified from Kachabira [48] and Dillla Zuria (in southeastern rift–valley agricultural landscapes of Gedeo Zone) ([6] (2 endemic species from each) of the southern Ethiopia. Except for one species, the remaining five endemic species were found in both HG and parkland systems (Figure 3(b)) of the present study area. Therefore, this study implies that the study area is currently dominated by indigenous species. However, Figure 3 indicates a potential increase in exotic species, suggesting a growing trend among farmers to introduce the exotic plants. This finding aligns with previous research by Legesse and Negash [48], Molla et al. [22], and Tesfay et al. [6], who reported similar distributions of indigenous, exotic, and endemic species in southern Ethiopia. Conversely, in Rwanda, exotic species have achieved dominance [45].

FIGURE 3Species origins in their total number and percentage (a), and distribution of species by habitat type (b) in the study area of Ethiopia. (a) Origin of the species to study area (Ethiopia). (b) Origin of the species across habitat types (Ethiopia).

**Figure:**
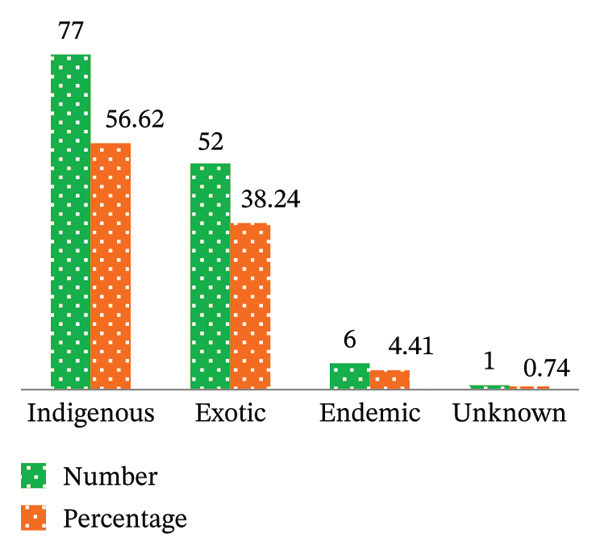
(a)

**Figure:**
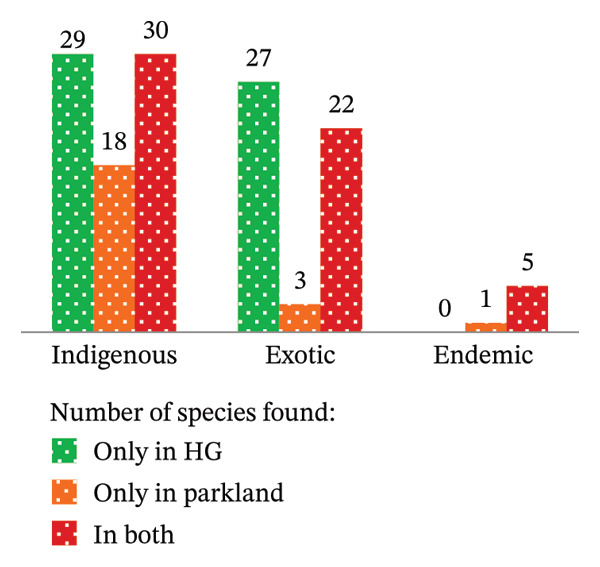
(b)

### 3.6. Species Diversity and Their Parametric Tests Across Three Districts in the Study Area

This study also analyzed plant species diversity in Ensaro, Mojana‐Worena, and Tarmaber districts. The results revealed significant changes among the three districts for HGs (Table 4).

**TABLE 4:** Diversity mean values of plant species for HGs and parkland across the three districts.

**Diversity indices**	**Mean values of diversity for HGs in three districts**			**Overall diversity values (mean)**
	**Ensaro (*n* = 20)**	**Moja (*n* = 20)**	**Tarmaber (*n* = 40)**	

Taxa‐S	6.90	8.85	10.35	114 (8.77)
Shannon‐H′	1.61	1.91	2.13	1.94
Evenness	0.86	0.86	0.88	0.86

	**Mean values of diversity for parkland in three districts**			
	**Ensaro (*n* = 8)**	**Moja (*n* = 8)**	**Tarmaber (*n* = 16)**	

Taxa‐S	7.33	6.78	6.67	79 (6.93)
Shannon‐H′	1.82	1.66	1.71	1.74
Evenness	0.92	0.86	0.89	0.88

	**Mean values of diversity for HGs and parkland**			
	**HGs (*N* = 80)**	**Parkland (*N* = 33)**		

Taxa‐S	8.77	6.93		136 (7.99)
Shannon‐H′	1.94	1.74		1.84
Evenness	0.86	0.88		0.87

#### 3.6.1. Species Diversity and Their Parametric Tests in HGs

The species richness of HGs varied significantly among the three districts, with Tarmaber exhibiting the highest total species count (86), followed by Mojana‐Werena (57) and Ensaro (42). When considering the average species richness per sample (taxa‐s), Tarmaber demonstrated the highest value (10.35), followed by Mojana‐Worena (8.85), whereas the least one was at Ensaro (6.90) (Table 4). Similarly, the highest the Shannon diversity (2.13) was recorded in Tarmaber District, followed by Mojana‐Werena (1.91), but the least one was at Ensaro District (1.61). Just like richness and diversity, despite minor differences, similar evenness trends were observed across the three districts. These all indicated a more diverse and equitably distributed plant community in Tarmaber District.

Multivariate analysis also confirmed significant differences for both taxa‐S (*p* = 0.008, df = 2, *F* = 5.119) and Shannon‐H′ (*p* = 0.001, df = 2, *F* = 7.824) in HGs among the three districts (Table 5). Notably, a significant difference was found between Ensaro and Tarmaber districts for both taxa‐S (*p* = 0.006) and Shannon‐H′ (*p* = 0.001), but not between other districts as shown in Table 6 (or supporting file 1). This suggests potentially better water availability and protection for HGs in Tarmaber. However, the evenness values in the three districts were relatively similar, with a slight increase in Tarmaber (Table 5). As a result, no significant difference was observed for evenness among the studied districts (*p* > 0.05) (Table 6).

**TABLE 5:** The multivariate test of the general linear model using the overall mean square scores for Shannon diversity indices of plant species in HGs and parkland agroforestry systems separately across the three districts with their degree of freedom, F values, and *p* values.

**Dependent variables**	**Mean square for HG data analysis (*N* = 80)**	**df**	***F***	**p** **value**

Taxa‐S	80.27	2	5.119	0.008
Shannon‐H′	1.84	2	7.824	0.001
Evenness	0.005	2	0.458	0.634

	**Mean square for parkland data analysis (*N* = 33)**			

Taxa‐S	1.313	2	0.200	0.820
Shannon‐H′	0.068	2	0.405	0.671
Evenness	0.006	2	0.735	0.488

	**Mean square for grand total of HGs and parkland data analysis (GN = 113)**			

Taxa‐S	116.568	1	8.255	0.005
Shannon‐H′	0.955	1	4.008	0.048
Evenness	0.004	1	0.377	0.540

**TABLE 6:** Pairwise comparison using a Tukey HDS of the post hoc tests for showing their significant difference between two of the three districts studied.

**Diversity indices**	**Mean ± SE for HGs**		**p** **value**	**Mean ± SE for parkland**		**p** **value**
	**Ensaro (*n* = 20)**	**Tarmaber (*n* = 40)**		**Ensaro (*n* = 9)**	**Tarmaber (*n* = 15)**	

Taxa richness	6.90 ± 0.83	10.35 ± 0.63	0.006	7.33 ± 0.85	6.67 ± 0.66	0.812
Shannon diversity	1.61 ± 0.14	2.13 ± 0.07	0.001	1.82 ± 0.14	1.71 ± 0.11	0.790
Evenness	0.86 ± 0.03	0.88 ± 0.01	0.835	0.92 ± 0.03	0.89 ± 0.02	0.761

	**Mean ± SE for HGs**			**Mean ± SE for Parkland**		
	**Ensaro**	**Moja**		**Ensaro**	**Moja (*n* = 9)**	

Taxa richness	6.90 ± 0.83	8.85 ± 0.91	0.270	7.33 ± 0.85	6.78 ± 0.85	0.890
Shannon diversity	1.61 ± 0.14	1.91 ± 0.11	0.128	1.82 ± 0.14	1.66 ± 0.14	0.659
Evenness	0.86 ± 0.03	0.85 ± 0.02	0.953	0.92 ± 0.03	0.87 ± 0.03	0.456

	**Mean ± SE for HGs**			**Mean ± SE for Parkland**		
	**Moja**	**Tarmaber**		**Moja**	**Tarmaber**	

Taxa richness	8.85 ± 0.91	10.35 ± 0.63	0.355	6.78 ± 0.85	6.67 ± 0.66	0.994
Shannon diversity	1.91 ± 0.11	2.13 ± 0.07	0.224	1.66 ± 0.14	1.71 ± 0.11	0.944
Evenness	0.85 ± 0.02	0.88 ± 0.01	0.634	0.87 ± 0.03	0.89 ± 0.02	0.795

*Note:* There is no difference for pairwise comparison between Moja and Ensaro (= Ensaro and Moja), Tarmaber and Ensaro (= Ensaro and Tarmaber), and Tarmaber and Moja (= Moja and Tarmaber) with the comparison made in Table 5 that is why they are omitted from listing them in the same table (also see supporting file 2).

#### 3.6.2. Species Diversity and Their Parametric Tests in Parklands

Similar to HGs, total species richness for parklands varied across districts. Ensaro, Mojana‐Worena, and Tarmaber had 29, 21, and 37 species, respectively (Table 4). However, the average species richness per sample (taxa‐s) showed an opposite trend compared to HGs. Ensaro (7.33) had a slightly higher value than both Mojana‐Werena (6.78) and Tarmaber (6.67) (Table 4). Furthermore, the Shannon‐H′ and evenness were also very slightly variable among the three districts. As a result, unlike HGs, there was no significant difference in taxa‐S (*p* = 0.820, df = 2, *F* = 0.200), Shannon‐H′ (*p* = 0.671, df = 2, *F* = 0.405), and evenness (*p* = 0.488, df = 2, *F* = 0.735) among the three districts for parklands (*p* ≥ 0.05) (Table 5). This lack of significant variation in species richness and diversity metrics for parklands could be attributed to the absence of fencing or protection for parkland plants, leading to similar levels of deforestation across all districts.

#### 3.6.3. Comparison and Parametric Tests Between HGs and Parklands

As depicted in Table 4, HGs exhibited significantly higher overall taxa‐S (mean = 8.77) and Shannon‐H’ (mean = 1.94) compared to parklands (taxa‐S = 6.93, H’ = 1.74). However, evenness (*J*) was slightly higher in parklands (*J* = 0.88) than in HGs (*J* = 0.86). Statistical analysis confirmed these differences. Pairwise parametric tests revealed significant variations between HGs and parklands for taxa‐S (*p* = 0.005, *F* = 8.255) and Shannon‐H’ (*p* = 0.048, *F* = 4.008), but not for evenness (*p* = 0.540, *F* = 0.377). This could be due to a combination of factors of higher moisture availability and fencing benefiting HGs, while potentially higher deforestation in the parklands might be caused by less moisture, free grazing, and tree felling by farmers for land preparation. Although HGs showed higher in Shannon diversity than parklands, the values for both systems were considered low, suggesting a disturbed ecological state. Generally, these findings suggest that though HGs have higher species richness and diversity, both systems exhibit relatively low overall diversity levels, indicating ecological disturbance. These findings are in line with the report by Guzo et al. [14].

Furthermore, Table 5 demonstrated that evenness remained consistent across districts for both HGs and parklands, suggesting an equitable distribution of species within these two ecosystem types. This is reflected by the absence of significant differences in evenness between districts for both HGs and parklands (*p* ≥ 0.05) (Table 5). Notably, all diversity indices in parklands lacked significant differences across districts (*p* ≥ 0.05) (Table 5). Overall, these results highlight the need for improved ecological management practices in both HGs and parklands to enhance biodiversity and ecosystem health in the study area.

### 3.7. Similarity of Plant Species Composition in HGs and Parkland

The similarity of the plant species composition between the two agroforestry practices was calculated using SSC. Of the total plant species (136), 57 and 22 species were identified only from the surveyed HGs and parkland, respectively. The remaining 57 plant species were identified from both HGs and parkland of the study area. Based on these data, the result of the SSC was 0.59 (or, 59%). This shows that the majority of the plant species growing in both HGs and parkland agroforestry practices were similar. In other words, the species found across all sampling plots of the agroforestry systems of the study area was homogenous or moderately distributed [6]. As a general standard, any two plant communities (of two categories) that have more than 50% similarity represent the same association [28, 49].

### 3.8. IVI for Structural Analysis of Woody Plant Species

Of the 114 and 79 plant species identified in HGs and parklands of the study area, respectively, only 23 and 24 woody species met the criteria for percentage of IVI analysis (Tables 5 and 6). These data of the species enabled the researcher to describe the vegetation structure using density, DBH, frequency distribution, height, and IVI [50]. Additionally, they were able to assess the relative ecological importance of each species [14]. For these species, therefore, the percentage of RF, RD, and RBA was calculated for each habitat (Tables 7 and 8) to determine the IVI for each species per land use (HG and parkland).

**TABLE 7:** The relative frequency (RF), Relative density (RD), relative basal area per hectare (RBA ha^−1^), and importance value index (IVI) of HGs of the study area.

Scientific name	RF	RD	RBA ha^−1^	IVI	Rank
*Rhamnus prinoides*	20.18	224.07	20.87	265.11	1^st^
*Eucalyptus globulus*	5.26	27.34	52.93	85.53	2^nd^
*Cupressus lusitanica*	2.63	6.53	8.06	17.22	3^rd^
*Croton macrostachyus*	7.89	1.98	3.57	13.45	4^th^
*Ziziphus spina-christi*	4.39	0.54	4.6	9.53	5^th^
*Schinus molle*	5.26	0.54	3.04	8.84	6^th^
*Grevillea robusta*	4.39	0.43	0.72	5.53	7^th^
*Millettia ferruginea*	4.39	0.45	0.62	5.45	8^th^
*Mangifera indica*	2.63	0.52	1.95	5.1	9^th^
*Cordia africana*	4.39	0.16	0.32	4.86	10^th^
*Olea europaea*	2.63	0.32	1.49	4.43	11^th^
*Citrus sinensis*	3.51	0.36	0.49	4.36	12^th^
*Moringa oleifera*	2.63	0.23	0.28	3.14	13^th^
*Vachellia albida*	2.63	0.07	0.19	2.89	14^th^
*Sesbania sesban*	2.63	0.07	0.11	2.81	15^th^
*Azadirachta indica*	2.63	0.07	0.09	2.79	16^th^
*Salix mucronata*	0.88	0.11	0.15	1.14	17^th^
*Schefflera abyssinica*	0.88	0.05	0.21	1.13	18^th^
*Citrus aurantium*	0.88	0.05	0.14	1.06	19^th^
*Hagenia abyssinica*	0.88	0.11	0.07	1.06	19^th^
*Vachellia seyal*	0.88	0.02	0.07	0.97	20^th^
*Erythrina brucei*	0.88	0.02	0.03	0.93	21^st^
*Casuarina cunninghamiana*	0.88	0.02	0.01	0.91	22^nd^

**TABLE 8:** The relative frequency (RF), relative density (RD), relative basal area per hectare (RBA ha^−1^), and importance value index (IVI) in parkland of the study area.

Scientific species	RF	RD	RBA ha^−1^	IVI	Rank
*Eucalyptus globulus*	12.66	68.13	61.54	142.32	1^st^
*Croton macrostachyus*	27.85	5.06	0.62	33.53	2^nd^
*Vachellia abyssinica*	8.86	3.05	6.22	18.13	3^rd^
*Ziziphus spina-christi*	5.06	3.77	8.63	17.46	4^th^
*Olea europaea*	11.39	0.77	2.12	14.28	5^th^
*Lantana Trifolia*	3.80	5.10	4.83	13.73	6^th^
*Erythrina brucei*	10.13	1.45	2.02	13.59	7^th^
*Vachellia senegal*	6.33	0.41	6.30	13.04	8^th^
*Juniperus procera*	10.13	1.14	0.70	11.97	9^th^
*Ficus sur*	8.86	0.29	0.48	9.63	10^th^
*Vachellia seyal*	7.59	0.56	0.89	9.05	11^th^
*Vachellia nilotica*	5.06	0.87	0.99	6.93	12^th^
*Vachellia albida*	3.80	0.60	2.43	6.83	13^th^
*Carissa spinarum*	6.33	0.35	0.10	6.78	14^th^
*Myrica salicifolia*	6.33	0.25	0.12	6.70	15^th^
*Vachellia bussei*	2.53	1.04	1.67	5.23	16^th^
*Mangifera indica*	2.53	0.25	0.12	2.90	17^th^
*Casuarina cunninghamiana*	2.53	0.12	0.14	2.80	18^th^
*Dombeya torrida*	2.53	0.04	0.01	2.58	19^th^
*Searsia retinorrhoea*	2.53	0.04	0.01	2.58	19^th^
*Hagenia abyssinica*	2.53	0.04	0.00	2.57	20^th^
*Ekebergia capensis*	1.27	0.12	0.02	1.41	22^th^
*Moringa oleifera*	1.27	0.02	0.04	1.32	23^th^
*Balanites aegyptiaca*	1.27	0.02	0.01	1.29	24^th^

#### 3.8.1. RF Distribution of Woody Species

The occurrence of woody species across the two agroforestry systems in the study area was different. *R. prinoides* (20.18%) was the most frequent, followed by *C. macrostachyus* (7.89%), *E. globulus* (5.26%), *Schinus molle* L. (5.26%), *Ziziphus spina-christi* (L.) Desf., *Grevillea robusta* R.Br., *M*. *ferruginea* and *Cordia africa* Lam., which occurred in 4.39% each of all sampled HGs (*n* = 40) (Figure 4(a), Table 7). In contrast, *Casuarina cunninghamiana* Miq., *E*. *brucei,* and *V. seyal* Del. were very rare, by occurring only at about a single sampled HG (Figure 4(a), Table 7). The remaining 13 woody species occurred in the range of 3.51 to 0.88% of the total sampled HGs of the study area (Table 7).

FIGURE 4The relative frequency distribution of woody plant species across home gardens (HGs) (a) and parklands (b) in the study area. (a) Woody species in HGs. (b) Woody species in parkland.

**Figure:**
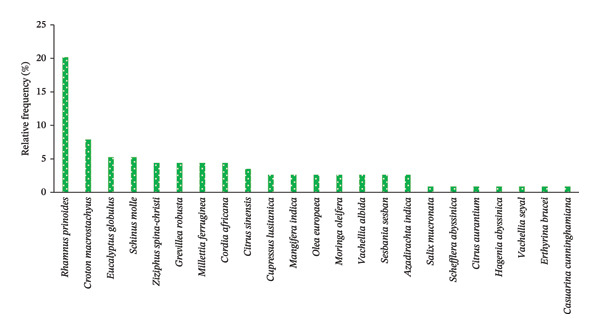
(a)

**Figure:**
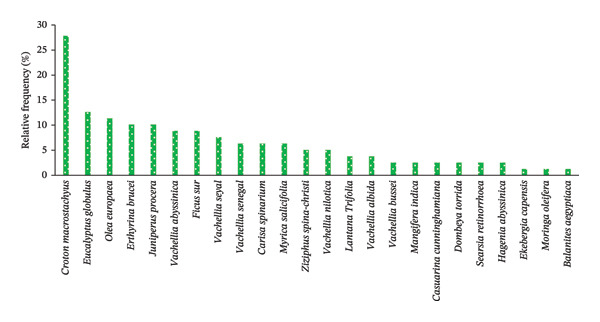
(b)

While considering parklands, the most frequently occurred plant species was *C. macrostachyus* (27.85%), followed by E. globules (12.66%), *Olea europaea* L. (11.39%), *E. brucei*, *J. procera* (10.13% each), *A. abyssinica*, *F. sure* (8.86%, each), and *A. seyal* (7.59%) (Figure 4(b), Table 8). This implies that those frequently occurred woody species including *C*. *macrostachyus* entirely occupied in 86.08% of the total sampled parklands (*n* = 32) of the study area. In contrast, *Ekebergia capensis* Sparrm, *Moringa oleifera* Lam., and *Balanites aegyptiaca* (L.) Del. occurred very rarely (1.27%, each), followed by *V. bussei* Harms ex Sjostedt., *Mangifera indica* L., *C. cunninghamiana*, *Dombeya torrida* (J. F. Gme1.) P. Bamps, *Searsia retinorrhoea* (Steud. ex Oliv.) Moffett, and *Hagenia abyssinica* (Brace) J.F.Gmel (2.53%, each) (Table 8). This finding is in contrast to the report of Molla et al. [22], who reported that *C. africana* was the most frequent woody species in the farmland of Wondo District of Southern Ethiopia.

Overall, *R. prinoides* and *C. macrostachyus* were the most frequently occurring species in HGs and parkland agroforestry systems, respectively, with other perspective common species, indicating their dominance and suitability of these species and demanding by the local people for the two agroforestry system of central Ethiopia.

#### 3.8.2. RD of Woody Species

RD is another important index to estimate the dominancy and suitability of the plant species to the existing agroecological and climatic conditions. Accordingly, the values of 23 woody species fulfilling the criteria for structural analysis of HGs are presented in Figure 5(a) and Table 7. The results indicate that *R. prinoides* had the highest density (224.07%), followed by *E. globulus* (27.34%) and *Cupressus lusitanica* Mill. (6.53%) (Figure 5(a), Table 7). Except *C. macrostachyus* (1.98%), the remaining woody species in HGs of the study area had less than or equal to 0.54%, each (ranging from 0.54 to 0.02%), indicating that the majority of these woody species (19 species) had very low density per ha in percentage (4.14 ha; on average, 0.414 ha each species had).

FIGURE 5The relative density per hectare of each woody plant species across home gardens (HGs). (a) and parklands (b) in the study area. (a) Woody species in HGs. (b) Woody species in parkland.

**Figure:**
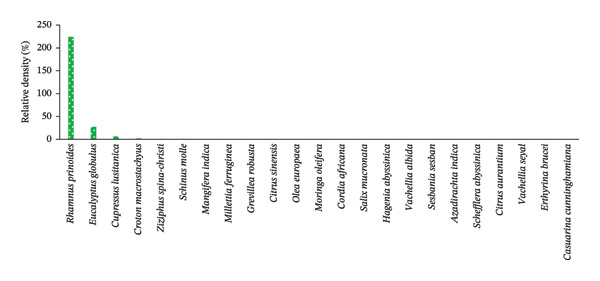
(a)

**Figure:**
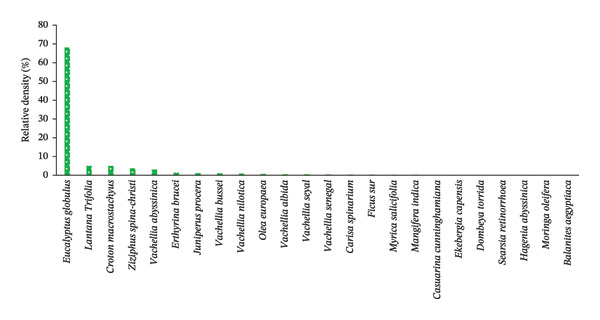
(b)

In the parkland, the values of 24 woody plant species fulfilling the criteria for structural analysis are presented in Figure 5(b) and Table 8. Accordingly, *E. globulus* had the highest density (68.13%), followed by *Lantana Trifolia L.* (15.38%), and *C. macrostachyus* (5.06%) (Figure 5(b), Table 7). On the contrary, 16 woody species had the least RD, less than 1%, ranging from 0.77 to 0.02%, of the total woody species found in parklands (*n* = 24). This indicates that *E. globulus* was the most dominant species growing in most parkland of central Ethiopia. Molla et al. [22] also reported as eucalyptus species (*E. camaldulensis*) had the highest density in farm of the southern Ethiopia.

#### 3.8.3. RBA of Woody Species

The values for 23 woody species fulfilling the criteria for the vegetation structural analysis of HGs in the study area are presented in Figure 6(a) and Table 7. The results denote that *E. globulus* had the highest BA in the study area (52.93%, 0.81 m^2^ ha^−1^), followed by *R. prinoides* (20.87%), *C. lusitanica* (8.06%), *Z. spina-christi* (4.6%), *C. macrostachyus* (3.57%), and *S. molle* (3.04), entirely representing 93.07% of the total BA of the HGs in the study area. The remaining 17 woody species for the structural analysis of the HGs totally accounted for 6.93% of the total BA of the HGs in the study area, implying that the 17 woody species represented less than 1% (only 0.41%, each species on average) of the total BA in HGs.

FIGURE 6The relative basal area (RBA) of each woody plant species across home gardens (HGs) (a) and parklands (b) in the study area. (a) Woody species in HGs. (b) Woody species in parkland.

**Figure:**
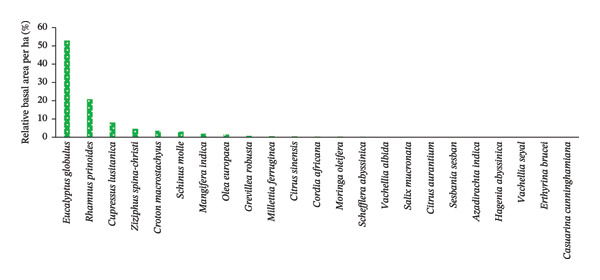
(a)

**Figure:**
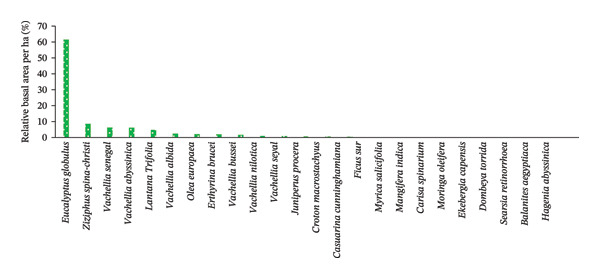
(b)

Similarly, 24 woody species fulfilling the criteria for vegetation structural analysis of parklands are presented in Figure 6(b) and Table 8. Of which, the most dominant species in terms of its highest BA was *E. globulus* (61.54%, 4.71 m^2^ ha^−1^) in the parkland of the study area, followed by *Z. spina-christi* (8.63%), *V. senegal* (L.) Wild. (6.30%), *V. abyssinica* (6.22%), *L*. *trifolia* (4.83%), and *V. albida* (2.43%). These six dominant species entirely covered 89.95% of the total BA of the parkland of the study area. The remaining 18 woody species of the parkland of the study area accounted for 10.05% BA (only 0.56%, each species, on average). The majority of these six dominant species, except *E. globulus* and *L. vulgaris*, in terms of their BA in the parkland were leguminous plant species, which are nitrogen‐fixing plants, thereby increasing the soil fertility of the parklands in the study area. Therefore, the scaling up of these leguminous species in all parkland (farmland) of Ethiopia is advisable and recommended. While *E. globulus* and *L. Trifolia* are currently most dominant and demanded by local farmers, they are not recommended to plant and scaling up them in parkland. This is because these particular species can negatively impact agricultural crops and change the soil physicochemical condition of the parkland. These species release substances through their leaves and roots that can be toxic to other plants and strongly compete for essential moisture and nutrients.

#### 3.8.4. IVI

As performed per HG and parkland land‐use system of the study area for RF, RD, and RBA to each woody species fulfilling the criteria for analyzing the vegetation structure, the values for IVI are presented in Tables 7 and 8 for HG and parkland, respectively. As a result, *R. prinoides* scored the highest IVI (IVI = 265.11), followed by *E. globules* (IVI = 85.53), *C. lusitanica* (IVI = 17.22), and *C. macrostachyus* (IVI = 13.45). This is because the highest RF (20.18%), RD (224.07%), and RBA (20.87%) were recorded for *R. prinoides*, followed by *E. globulus* and *C. lusitanica*. This suggests *R. prinoides*, *E. globulus*, and *C. lusitanica* are the most abundant and ecologically important woody species (Table 7). This also indicates that those species were more preferred by local communities, adaptable, and successful species in HGs of the studied agroecological zones. This result is almost similar to the finding of Woldeyohannes and Moges [26]. Other studies conducted elsewhere in Ethiopia also concluded that species that have high IVI values could be used as indicators for ecologically important woody species [14, 19, 50]. However, *V*. *seyal*, *E*. *brucei*, and *C*. *cunninghamiana* had the lowest IVI, respectively, implying their less adaptability and suitability in the ecological zone. Those species showing higher IVI values need a close monitoring management; however, the species having lower IVI values require high conservation efforts [51]. In contrast to this finding, the study conducted in southern Ethiopia’s HG systems prioritized *C*. *africana*, *Coffea arabica* L., *Persea americana* Mill, and *M*. *indica*, respectively [48].

Regarding the IVI of the species in parkland, the most abundant woody species in the parkland of the study area was *E. globulus* (IVI = 142.32), followed by *C. macrostachyus* (IVI = 33.53), *V. abyssinica* (IVI = 17.46), and *O*. *europaea* (IVI = 14.28). Despite *E. globulus *plantations altering certain soil physicochemical properties [52], farmers favored planting it in their parkland. This preference may be attributed to its multiple uses and the scarcity of alternative tree products, such as timber, farming tools, and fuel wood, from other local forested areas. As noted by Alemayehu and Melka [53], rising wood prices and increased demand have led smallholders to convert farmland into eucalyptus plantations. Additionally, limited land availability in the study area may contribute to this trend.

## 4. Conclusions

Based on the major findings, this study concludes that agroforestry systems—particularly HGs—play a critical role in maintaining plant diversity and ecological stability in the NSZ. The markedly higher species richness and diversity observed in HGs compared to parklands demonstrate their importance as key reservoirs of woody plant diversity and as multifunctional systems that meet farmers’ livelihood needs while supporting conservation goals. The dominance of a few species with high IVIs, such as *R. prinoides*, *Croton macrostachyus*, and *Eucalyptus globulus* in HGs, and *C. macrostachyus*, *V. abyssinica*, and *O. europaea* in parklands, underscores their central ecological and livelihood roles, while also signaling the risk of structural simplification if management continues to favor a narrow set of species. The prevalence of native species (over 60%) underscores the contribution of agroforestry to conserving indigenous flora, while the increasing integration of exotic species points to the need for careful management to balance livelihood benefits with potential ecological risks. Species richness and diversity vary markedly across districts, with Tarmaber consistently demonstrating distinct patterns. These differences underscore the strong influence of localized management practices and agroecological conditions on agroforestry outcomes. However, the observed decline in agroforestry practices, driven by insecure land ownership, population pressure, limited awareness, and gaps in scientific evidence, poses a serious threat to these benefits. Therefore, the present study documented the composition, diversity, and structural importance of woody species in HGs and parklands; therefore, it provides a strong empirical foundation for conservation planning and for developing more effective, locally appropriate land management strategies. Overall, these findings imply that strengthening land tenure security, enhancing community awareness, and promoting evidence‐based extension services tailored to district‐level realities are essential to sustain and scale up agroforestry systems.

## Author Contributions

The author conceptualized, executed, and revised the research, including data collection, analysis, manuscript preparation, and funding acquisition.

## Funding

This research was funded by DBU, with Grant No. 386‐08‐01.

## Disclosure

The university or other individuals had no involvement in the study design, data collection, analysis, writing, or revision of the manuscript.

## Conflicts of Interest

The author declares no conflicts of interest.

## Supporting Information

Additional supporting information can be found online in the Supporting Information section.

## Supporting information


**Supporting Information 1** Supporting file 1: Pairwise comparisons for home garden plot data using Turkey HSD of post hoc tests of genera linear model.

**Supporting Information 2** Supporting file 2: Pairwise comparisons for the parkland plot data using Tukey HSD of post hoc tests of general linear model.

## Figures and Tables

**Figure:**
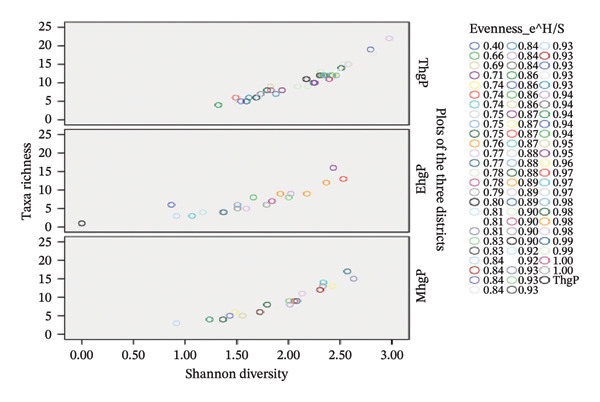
(a)

**Figure:**
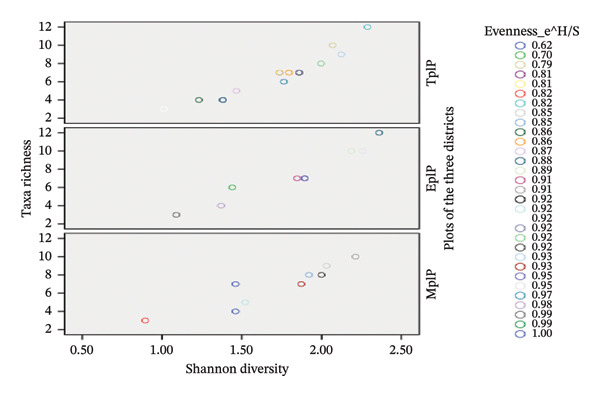
(b)

**Figure:**
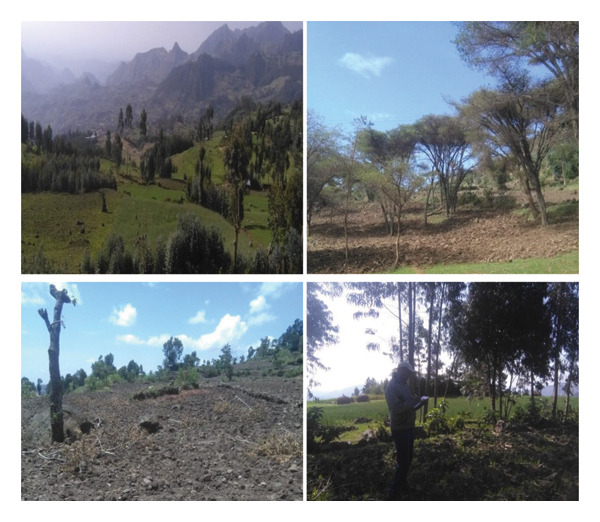
(a)

**Figure:**
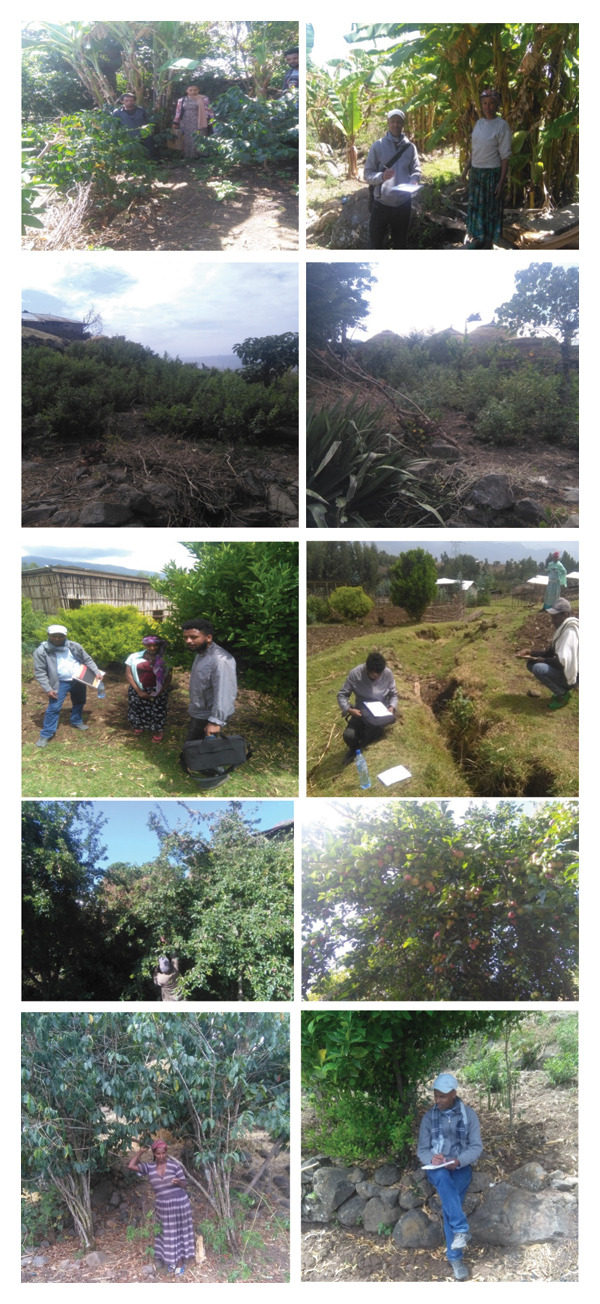
(b)

## Data Availability

The data that support the findings of this study are available from the corresponding author upon reasonable request.

## Appendix A

**Table A1:** List of plant species with their botanical and local names, growth form, and uses identified from HG and Pl of the study area.

Scientific name	Local name	Family name	Growth form	Uses of the plant	Origin	Found in	Voucher number
*Afromorus mesozygia* (Stapf ex A.Chev.) E.M.Gardner.	Enjori	Moraceae	Shrub/tree	Fw, Co, Fr, Sh	Indigenous	HG	111AA
*Agave sisalana* Perrine ex Engl.	Qacha	Agavaceae	Herb	R, Md, SC	Exotic	HG, Pl	038 AA
*Allium cepa* L.	Qey Shinkurt	Alliaceae	Herb	F, Md, Sp,	Exotic	HG, Pl	012 AA
*Allium sativum* L.	Nech shinkurt	Alliaceae	Herb	F, Md, Sp	Exotic	HG	044 AA
*Allophylus abyssinicus* (Hachst.) Radlk	Embus	Sapindaceae	Tree	Fw, Ch, Fe	Indigenous	HG, Pl	052 AA
*Aloe trichosantha* Berger	Eret (wondie)	Aloaceae	Herb	Md, SC	Indigenous	HG, Pl	053 AA
*Arundo donax* L.	Shenbeqo	Poaceae	Herb	Or, Co, Fu	Indigenous	HG	024 AA
*Asparagus africanus* Lam.	Serity	Asparagaceae	Shrub	Lf, Md	Indigenous	HG	079AA
*Azadirachta indica* A. Juss	Nim/Kinin	Meliaceae	Tree	Md, Or, Sh, Inc, WB	Exotic	HG	022 AA
*Balanites aegyptiaca* (L.) Del.	Jemo| bedeno	Balanitaceae	Tree	F, Fr, O (fruit)	Indigenous	Pl	114AA
*Bersama abyssinica* Fressen	Azamir	Melianthaceae	Shrub/tree	Fw, Md, BF, Lf	Indigenous	Pl	128AA
*Beta vulgaris* L. var. cicla	Qosta	Chenopodiaceae	Herb	F, IS	Exotic	HG	119 AA
*Brassica oleracea* L.	Yeabesha gomen	Brassicaceae	Herb	F, IS	Indigenous	HG	066AA
*Calotropis procera* (Aiton) W. T. Aiton	Yeregna qolo	Apocynaceae	Shrub	FW. Md, Fi	Indigenous	Pl	133AA
*Calpurnia aurea* (Alt.)Benth	Digita	Fabaceae	Shrub/tree	Fw, Fe	Indigenous	HG, Pl	084 AA
*Capparis tomentosa* Lam.	Gumero	Capparidaceae	Shrub	Md, Fe, Lf, Fw	Indigenous	Pl	115AA
*Capsicum annum* L.	Berberie	Solanaceae	Herb	F, Md	Exotic	HG, Pl	010AA
*Carica papaya* L.	Papaya	Caricaceae	Herb	F, IS	Exotic	HG	033 AA
*Carissa spinarum* L.	Agam	Apocynaceae	Shrub	Fw, F, Md	Indigenous	HG, Pl	006 AA
*Casuarina cunninghamiana* Miq.	Shewshewe	Casuarinaceae	Tree	Or, SC, SF, Co, Fw, Ch	Exotic	HG	027AA
*Catha edulis* (Vahl) Forssk. ex Endl.	chat	Celastraceae	Shrub	SD, Md, Fw	Indigenous	HG	051AA
*Celtis africanus* Burm. f.	Kewet	Ulmaceae	Tree	Fw, SF, Fd, Lf	Indigenous	HG	092AA
*Cenchrus geniculatus* Thunb	Yewisha sar	Poaceae	Herb	Fd, SC	Indigenous	Pl	123AA
*Cenchrus purpureus* (Schumach.) Morrone.	Yezhone sar	Poaceae	Herb	Fd, Fr, Th, SC	Exotic	HG, Pl	081AA
*Cicer arietinum* L.	shibira	Fabaceae	Herb	Fd, F, SF	Exotic	Pl	122AA
*Citrus aurantiifolia* (christm.) Swingle	Lomi	Rutaceae	Shrub	F, Md, IS	Exotic	HG, Pl	009AA
*Citrus aurantium* L.	komitate lomi	Rutaceae	Shrub	Fw, F, Md	Exotic	HG	136 AA
*Citrus medica* L.	Tiringo	Rutaceae	Shrub	F, Md	Exotic	HG	035 AA
*Citrus reticulate* Blanco	Menderin	Rutaceae	Tree	F, IS	Exotic	HG	099 AA
*Citrus sinensis* (L.) Obs.	Birtukan	Rutaceae	Shrub	F, J, IS	Exotic	HG, Pl	016 AA
*Clutia abyssinica* Jaub. & Spach.	Fiyelefej	Clusiaceae	Shrub	Fd	Indigenous	HG	095 AA
*Coffea arabica* L.	Buna	Rubiaceae	Shrub/tree	J, Md, IS	Indigenous	HG, Pl	013AA
*Cordia africana* Lam.	Wanza	Boraginaceae	Tree	FW, Co, Fd, IS, SF,Md	Indigenous	HG	005 AA
*Corymbia citriodora* (Hook.) K.D. Hill & L.A.S. Johnson.	Shito Bahrzaf	Myrtaceae	Tree	Fw, Co	Exotic	HG	034 AA
*Croton macrostachyus* Del.	Bisana	Euphorbiaceae	Shrub/tree	Fw, Md, SC, Fu, Fd, SF	Indigenous	HG, Pl	019AA
*Cucurbita pepo* L.	Duba	Cucurbitaceae	Climber	F, Md	Exotic	HG	098AA
*Cupressus lusitanica* Mill.	yeferenj tsid	Cupressaceae	Tree	Fw, Or, Co	Exotic	HG, Pl	054AA
*Cymbopogon citratus* (DC.) Stapf.	Tej sar	Poaceae	Herb	Or, Ce, Md	Exotic	HG	073AA
*Cynodon dactylon* (L.) Pers.	Serdo sar	Poaceae	Herb	Fd, SC	Indigenous	Pl	117AA
*Cyperus alatus* (Nees) F. Muell	Engcha tinshua	Cyperaceae	Herb	Fd, Fr	Indigenous	Pl	129AA
*Cyperus fischerianus* A. Rich		Cyperaceae	Herb	Fd, Fr	Indigenous	HG	061AA
*Cyperus pauper* H ochft. ex A Rich	Qetema	Cyperaceae	Herb	Fd, Ce, Th, Mt, LB	Indigenous	HG	068AA
*Dacus carota* L.	Carrot	Apiaceae	Herb	F, IS	Exotic	HG	057 AA
*Delonix regia* (Boj. Ex Hook.) Raf.	Dire dawa zaf	Caesalpinioideae	Tree	FW, BF, Sh, Or	Exotic	HG	097 AA
*Discopodium penninervium* Hochst.	Ameraro	Solanaceae	Shrub	SC, Fe, Md	Indigenous	HG, Pl	109AA
*Dodonaea viscosa subsp. angustifolia* (L.f.) J.G.West	Kitikita	Sapindaceae	Shrub/tree	FW, SC, Fd, Fe, Md	Indigenous	HG	078AA
*Dombeya torrida* (J. F. Gme1.) P. Bamps	wulkfa	Sterculiaceae	Shrub	FW, SC, Fd, Md	Indigenous	Pl	132AA
*Dovyalis abyssinica* (A. Rich) Warb.	Koshim	Flacourtiaceae	Shrub/tree	FW, F, LF, Md, BF	Indigenous	HG, Pl	023AA
*Ekebergia capensis* Sparrm.	Lol	Meliaceae	Tree	FW, Co, FT, BF, Md	Indigenous	HG, Pl	105AA
*Ensete ventricosum* (Welw.) Cheesman	Koba/enset	Musaceae	Herb	BR, BB, F, Md, Th, IS	Indigenous	HG, Pl	003 AA
*Entada abyssinica* Steud ex A. Rich.	kentafa/qontir	Fabaceae	Shrub/tree	Fw, Ch, LF, Md	Indigenous	HG	090 AA
*Eragrostis cilianensis* (All.) Vign. ex Janchen	Sar	Poaceae	Herb	Fd, Fr	Indigenous	HG	104 AA
*Eragrostis tef* (Zucc.) Trotter	Tef	Poaceae	Herb	F, Fd, Md, IS	Endemic	HG, Pl	091AA
*Erythrina abyssinica* Lam. ex DC.	Korch	Fabaceae	Shrub	Fw, Ut, BF, Lf	Indigenous	HG	110AA
*Erythrina brucei* Schweinf	Ergofit/Korch	Fabaceae	Tree	Fw, Md, Lf, BF	Endemic	HG, Pl	040 AA
*Eucalyptus camaldulensis* Dehnh.	Qey bahirzaf	Myrtaceae	Tree	Fw, Ch, Co, Fe,	Exotic	HG, Pl	021 AA
*Eucalyptus globulus* Labill	Nech bahirzaf	Myrtaceae	Tree	Fw, Ch, Co, LF, Fe, Md, Fu	Exotic	HG, Pl	025 AA
*Euclea racemosa* Murr.	Dedeho	Ebenaceae	Shrub/tree	Fw, Lf, FT, Or	Indigenous	HG, Pl	039 AA
*Euphorbia abyssinica* Gmel.	kulkual	Euphorbiaceae	Shrub	Fw, Co, LF, Fu	Indigenous	Pl	125AA
*Festuca abyssinica* Hochst. ex A. Rich.	Guasa sar	Poaceae	Herb	Fd, Ce, Mt, R	Indigenous	HG	112AA
*Ficus carica* L.	Beles/Etsebeles	caciae	Shrub/tree	F, Md	Exotic	HG, Pl	050 AA
*Ficus sur* Forssk.	Shola	Moraceae	Tree	Co, F, Ce, Sh, Fu	Indigenous	HG, Pl	087AA
*Gossypium barbadense* L.	Tit	Malvaceae	Shrub	RMC	Indigenous	HG	049 AA
*Grevillea robusta* R. Br.	Grevilea/meka	Proteaceae	Tree	Fw, Ch, Co, SC, BF	exotic	HG, Pl	041AA
*Grewia ferruginea* Hochst. ex A. Rich	Lenquata	Tiliaceae	Shrub	Fw, LF, Fe, Md,	Indigenous	Pl	042AA
*Grewia villosa*	Lenquata	Tiliaceae	Shrub	Fw, Co, Md, F	Indigenous	HG, Pl	043 AA
*Gymnanthemum amygdalinum* (Delile) Sch.Bip. ex Walp	Girawa	Asteraceae	Shrub/tree	FW, Ch, Md, F, LF, Fd. SC, Mu	Indigenous	HG, Pl	045 AA
*Gymnosporia senegalensis* (Lam.) Loes	Yedega/Nech atat	Celastraceae	Shrub/tree	Fw, Md, Lf, Fe	Indigenous	Pl	116 AA
*Hagenia abyssinica* (Brace) J.F.Gmel	Koso	Rosaceae	Tree	Fw, Co, Mu, Md, GM, SF, SC	Indigenous	HG, Pl	065AA
*Hibiscus micranthus* Hochst. ex A. Rich	Nacha	Malvaceae	Shrub	Fw, MR	Indigenous	HG	080AA
*Hordeum vulgaris* L.	Gebs	Poaceae	Herb	F, Fd, Md	Indigenous	HG, Pl	083 AA
*Hypericum revolutum* Vahl	Amija	Hypericaceae	Shrub/tree	Fw, BF, CO, SC	Indigenous	Pl	131AA
*Impatiens rothii* Hook. f.	Insosla	Balsaminaceae	Herb	Dye	Endemic	Pl	130AA
*Jacaranda mimosifolia* D. Don	Yetemenja zaf	Bignoniaceae	Tree	BF, Co, FW	Exotic	HG	007 AA
*Juniperus procera* Hochst. ex Endl.	Ye‐abesha tsid	Cupressaceae	Tree	Co, Or	Indigenous	HG, Pl	075AA
*Justicia schimperiana* (Hochst. ex Nees) T. Anders.	Sensel	Acanthaceace	Shrub	FW, LF	Indigenous	HG	094 AA
*Lactuca sativa* L.	Selata	Asteraceae	Herb	F, IS	Exotic	HG	101 AA
*Lagenaria siceraria* (Molina) Slandl	Qil	Cucurbitaceae	Climber	Ct	Indigenous	HG	067 AA
*Laggera tomentosa* (Sch. Bip. ex A. Rich.) Oliv. & Hiern	Kesie/keskesso	Asteraceae	Shrub	Fw, Md, FuC	Endemic	HG, Pl	108 AA
*Lantana Trifolia* L.	Qimo|yeregnaqolo	Anacardiaceae	Shrub/Tree	FW, FT, F, Md	Indigenous	Pl	113AA
*Maesa lanceolata* Forssk.	Kelawa	Myrsinaceae	Tree	Fw, BB, Md, LF, Fe	Indigenous	HG, Pl	086 AA
*Malus sylvestris* Mill.	Pom/Apple	Rosaceae	Tree	FW, F, J, IS, SC,Or, Bf	Exotic	HG, Pl	055 AA
*Mangifera indica* L.	Mango	Anacardiaceae	Shrub	FW, F, J, Fd, BF, Sh, Or, SC	Exotic	HG, Pl	017AA
*Melia azedarach* L.	Melia	Meliaceae	Tree	Fw, Co, Or, Md, BF,Sh	Exotic	HG	106AA
*Millettia ferruginea* (Hochst.) Bak.	Birbira	Fabaceae	Tree	Fw, HT, Ut, FP, Sh	Endemic	HG, Pl	011AA
*Moringa oleifera* Lam.	Shiferaw	Moringaceae	Tree	FW, Fd, Md, Sp, SC, Sh, LF, BF, WP, WB	Exotic	HG, Pl	032 AA
*Musa × paradisiaca* L.	Muz	Musaceae	Herb	F, Md	Indigenous	HG, Pl	059AA
*Myrica salicifolia* Hochst. ex A. Rich	Shinet	Myricaceae	Shrub	Fw, Co, Md	Indigenous	Pl	126AA
*Nicotiana tabacum* L.	Timbaho	Solanaceae	Shrub	Md, To	Exotic	HG	134AA
*Ocimum americanum* L.	Besobila	Lamiaceae	Herb	F, Md	Exotic	HG	082AA
*Ocimum basilicum* L	Yezinjero Besobila	Lamiaceae	Herb	Fd, BF	Unknown	HG	004 AA
*Ocimum lamiifolium* Hochst. ex Benth	Damakesie	Lamiaceae	Shrub	FW, Md, Sp, BF	Indigenous	HG	047 AA
*Olea europaea* L.	woira 1	Oleaceae	Tree	Fw, Ch, Co, P, Md, Ce	Indigenous	HG, Pl	046 AA
*Persea americana* Mill.	Avocado	Lauraceae	Tree	F, Sh, O, IS	Exotic	HG, Pl	088 AA
*Phytolacca dodecandra* L. Herit.	Endod	Phytolaccaceae	Climber	WC, Md	Indigenous	HG	037 AA
*Pisum sativum* L.	Ater	Fabaceae	Herb	F, Fd, SF < IS	Indigenous	Pl	127AA
*Premna schimperi* Engl.	Chocho	Lamiaceae	Shrub/tree	Fw, Ch, Md, Fe	Indigenous	HG	074AA
*Prunus persica* (L.) Batsch.	Kock	Rosaceae	Tree	Fw, F, IS	Exotic	HG	107 AA
*Psidium guajava* L.	Zeitun	Myrtaceae	Shrub	F, Fw, IS	Exotic	HG	085 AA
*Raphanus raphanistrum* L.	Qeysir	Brassicaceae	Herb	F, IS	Exotic	HG	100AA
*Rhamnus prinoides* L. Heril	Gesho	Rhamnaceae	Shrub	LB, Md, Fl, FW, IS	Indigenous	HG, Pl	001 AA
*Ricinus communis* L.	Gulo	Euphorbiaceae	Shrub	Md, O	Indigenous	HG, Pl	014 AA
*Rosa abyssinica* Lindley	Tsigereda	Rosaceae	Shrub	Fw, F, Md, Lf, Or	Indigenous	HG, Pl	036AA
*Rubus apetalus* Poir	Prime	Rosaceae	Shrub	Fw, F	Exotic	HG	135 AA
*Rumex nepalensis* Spreng.	Tult	Polygonaceae	Shrub	Md	Exotic	HG, Pl	071AA
*Rumex nervosus* Vahl	Enbuacho	Polygonaceae	Shrub	Md, SC,	Endemic	HG, Pl	070AA
*Ruta chalepensis* L.	Tenadam	Rutaceae	Herb	Md, Fl	Indigenous	HG, Pl	002 AA
*Saccharum officinarum* L.	Shenkora ageda	Poaceae	Herb	F, SP, IS	Exotic	HG, Pl	060AA
*Salix mucronata* Thunb	Ahaya	Salicaceae	Tree	Fw, FT, Md	Indigenous	HG	069AA
*Salvia rosmarinus* Scheid	Yesiga metibesha	Lamiaceae	Shrub	Or, MR	Exotic	HG, Pl	048 AA
*Schefflera abyssinica* (Hockst. ex A. Rick) Harms	Cheferie/Gitem	Araliaceae	Tree	Fw, FT	Indigenous	HG	015 AA
*Schinus molle* L.	Kundo berberie	Anacardiaceae	Tree	Fw, Sh	Exotic	HG	008 AA
*Searsia retinorrhoea* (Steud. ex Oliv.) Moffett	Tilem	Anacardiaceae	Shrub/tree	Fw, FT	Indigenous	Pl	118AA
*Senna didymobotrya* (Fresen.) Irwin & Barneby	tufa/gufa	Fabaceae	Shrub	FW, Md, SC, Mu	Indigenous	HG, Pl	120AA
*Sesbania sesban* (L.) Merr.	Turmatur	Fabaceae	Shrub/tree	Fd, SF, SF, BF	Indigenous	HG, Pl	018 AA
*Sida tenuicarpa* Vollesen	chifrig	Malvaceae	Shrub	WP, Ut	Exotic	HG	102 AA
*Solanum lycopersicum* L	Timatim (exotic)	Solanaceae	Herb	F (fruit), IS	Exotic	HG, Pl	089 AA
*Solanum tuberosum* L.	Dinch	Solanaceae	Herb	F (tuber)	Exotic	HG	056AA
*Sorghum bicolor* (L.) Moench	Mashila	Poaceae	Herb	F (seeds), Fd	Indigenous	HG	096 AA
*Stephania abyssinica* (Dillon & A.Rich.)Walp.	Engocht |kelela	Menispermaceae	Climber	Md (all parts)	Exotic	Pl	124AA
*Terminalia leiocarpa* (DC.) Baill	Kewot	Combretaceae	Tree	Fw, Md	Indigenous	HG	093AA
*Trigonella* *foenumgraceum* L.	Abish	Fabaceae	Herb	F, Fd, Md, SF, IS	Exotic	Pl	121AA
*Triticum aestivum* L.	Sindie	Poaceae	Herb	F, Fd, Md, IS	Exotic	HG, Pl	077AA
*Vachellia abyssinica* Hochst. ex Benth.	Yeabesha girar	Fabaceae	Tree	Fw, Ch, SF, Fr	Indigenous	HG	026AA
*Vachellia albida* Del.	Girar	Fabaceae	Tree	Fr, Ch, SF, Fr, Mu	Indigenous	HG, Pl	031AA
*Vachellia brevispica Harms*	Qontir/ketefa	Fabaceae	Shrub/tree	Fw, Ch, SF, Fr	Indigenous	Pl	063AA
*Vachellia bussei* Harms ex Sjostedt.	Girar	Fabaceae	Shrub	Fw, Chl, Tn,	Indigenous	HG	029 AA
*Vachellia decurrens* Wild	Akacha/Mimosa	Fabaceae	Shrub	Fw, Ch, CO, SF, Or	Exotic	HG	072 AA
*Vachellia nilotica* (L.) Willd ex Del.	Cheba girar	Fabaceae	Tree	Fw, Ch, SF, Fd, Fc	Indigenous	Pl	064AA
*Vachellia saligna* (Labill.) Wendl	Akacha saligna	Fabaceae	Shrub/tree	Fw, SF, Fd, Lf	Exotic	HG	103 AA
*Vachellia senegal* (L.) Wild	Kosele girar	Fabaceae	Tree	Ga, Md, Fw, Ch, SF	Indigenous	HG, Pl	062AA
*Vachellia seyal* Del.	Wacho girar	Fabaceae	Tree	Fw, Ch, SF	Indigenous	HG, Pl	028 AA
*Vachellia tortilis* (Forssk.) Hayne.	Deweni girar	Fabaceae	Tree	Fw, Ch, Fd, SF	Indigenous	HG	076AA
*Vicia faba* L.	Baqela	Fabaceae	Herb	F,Fd, Fr, SF, IS	Exotic	HG, Pl	058AA
*Zea mays* L.	Beqolo	Poaceae	Herb	F, Fd, IS	Exotic	HG, Pl	030 AA
*Ziziphus spina-christi* (L.) Desf.	Qurqura	Rhamnaceae	Shrub/tree	F, Fw, Ch, Fd, Fe	Indigenous	HG, Pl	020 AA

*Note:* Pl, Parkland; Fw, firewood; Ch, charcoal; Fd, fodder; Fr, forage; Tn, tannin; Fe, fence; Md, medicine; R, rope; GA, Gum Arabic source; Or, ornamental; Sh, shade; F, food; Th, thatching; Mu, mulch; O, oil; Ce, ceremony; Sp, spice; Co, construction; Fl, favoring; S, sugar; J, juice; Ct, container; Mt, mattress; Fu, furniture; Inc, insecticide; dye, as cosmetic for coloring the hands and feet; RMC, raw material for making clothes; RLB, raw material for making a local boat; Fi, Fiber; FUC, fumigant and cleansing milk container; To, tobacco.

Abbreviations: BB, bread baking; BF, bee forage; Bh, bee hives; BW, butter wrapping; FC, flue curing; FT, farm tools; GM, green manure; HG, home garden; Is, income sources; LF, live fence; MR, meat roasting; Sc, soil conservation; SD, stimulant drug; Sf, soil fertility; WB, wind break WP, water purification.
