# Supplementary material for: Analysis of Plant Diversity and Importance Value Index in Central Ethiopian Agroforestry Systems
Source: Scientifica (Cairo). 2026 Apr 15;2026:9959255. doi: 10.1155/sci5/9959255 (PMC13080502; doi:10.1155/sci5/9959255)
Supplement: Supplementary file 1 — Supporting Information 1 Supporting file 1: Pairwise comparisons for home garden plot data using Turkey HSD of post hoc tests of genera linear model. [file SCI5-2026-9959255-s002.docx]

Supplementary file 1: Pairwise comparisons for home garden plot data using Turkey HSD of Post Hoc Tests of genera linear model

| Dependent Variable | District (I) | | District (J) | Mean Difference (I-J) | Std. Error | Sig. | 95% Confidence Interval | |
| --- | --- | --- | --- | --- | --- | --- | --- | --- |
|  |  |  |  |  |  |  | Lower Bound | Upper Bound |
| Taxa richness | Ensaro | Mojana-Wodera | | -1.95 | 1.252 | .270 | -4.94 | 1.04 |
|  |  | Tarmaber | | -3.45^*^ | 1.084 | .006* | -6.04 | -.86 |
|  | Mojana-Wodera | Ensaro | | 1.95 | 1.252 | .270 | -1.04 | 4.94 |
|  |  | Tarmaber | | -1.50 | 1.084 | .355 | -4.09 | 1.09 |
|  | Tarmaber | Ensaro | | 3.45^*^ | 1.084 | .006* | .86 | 6.04 |
|  |  | Mojana-Wodera | | 1.50 | 1.084 | .355 | -1.09 | 4.09 |
| Shannon diversity | Ensaro | Mojana-Wodera | | -.3015 | .15331 | .128 | -.6678 | .0649 |
|  |  | Tarmaber | | -.5228^*^ | .13277 | .001* | -.8401 | -.2055 |
|  | Mojana-Wodera | Ensaro | | .3015 | .15331 | .128 | -.0649 | .6678 |
|  |  | Tarmaber | | -.2214 | .13277 | .224 | -.5387 | .0959 |
|  | Tarmaber | Ensaro | | .5228^*^ | .13277 | .001* | .2055 | .8401 |
|  |  | Mojana-Wodera | | .2214 | .13277 | .224 | -.0959 | .5387 |
| Evenness_e^H/S | Ensaro | Mojana-Wodera | | .0093 | .03152 | .953 | -.0660 | .0846 |
|  |  | Tarmaber | | -.0157 | .02729 | .835 | -.0809 | .0496 |
|  | Mojana-Wodera | Ensaro | | -.0093 | .03152 | .953 | -.0846 | .0660 |
|  |  | Tarmaber | | -.0249 | .02729 | .634 | -.0902 | .0403 |
|  | Tarmaber | Ensaro | | .0157 | .02729 | .835 | -.0496 | .0809 |
|  |  | Mojana-Wodera | | .0249 | .02729 | .634 | -.0403 | .0902 |

***Note****: The error term is the mean square = 0.01, * the mean difference is significant at 0.05 level*
