# Supplementary material for: Analysis of Plant Diversity and Importance Value Index in Central Ethiopian Agroforestry Systems
Source: Scientifica (Cairo). 2026 Apr 15;2026:9959255. doi: 10.1155/sci5/9959255 (PMC13080502; doi:10.1155/sci5/9959255)
Supplement: Supplementary file 2 — Supporting Information 2 Supporting file 2: Pairwise comparisons for the parkland plot data using Tukey HSD of post hoc tests of general linear model. [file SCI5-2026-9959255-s001.docx]

Supplementary file 2: Pairwise comparisons for parkland plot data using Tukey HSD of Post-Hoc Tests of general linear model

| Dependent Variable | (I) plots | (J) plots | Mean Difference (I-J) | Std. Error | Sig. | 95% Confidence Interval | |
| --- | --- | --- | --- | --- | --- | --- | --- |
|  |  |  |  |  |  | Lower Bound | Upper Bound |
| Taxa_S | EplP | MplP | .56 | 1.208 | .890 | -2.42 | 3.53 |
|  |  | TplP | .67 | 1.080 | .812 | -2.00 | 3.33 |
|  | MplP | EplP | -.56 | 1.208 | .890 | -3.53 | 2.42 |
|  |  | TplP | .11 | 1.080 | .994 | -2.55 | 2.77 |
|  | TplP | EplP | -.67 | 1.080 | .812 | -3.33 | 2.00 |
|  |  | MplP | -.11 | 1.080 | .994 | -2.77 | 2.55 |
| Shannon_H | EplP | MplP | .169711 | .1935109 | .659 | -.307346 | .646768 |
|  |  | TplP | .113644 | .1730814 | .790 | -.313048 | .540337 |
|  | MplP | EplP | -.169711 | .1935109 | .659 | -.646768 | .307346 |
|  |  | TplP | -.056067 | .1730814 | .944 | -.482759 | .370626 |
|  | TplP | EplP | -.113644 | .1730814 | .790 | -.540337 | .313048 |
|  |  | MplP | .056067 | .1730814 | .944 | -.370626 | .482759 |
| Evenness_e^H/S | EplP | MplP | .049467 | .0408266 | .456 | -.051182 | .150115 |
|  |  | TplP | .025842 | .0365164 | .761 | -.064181 | .115865 |
|  | MplP | EplP | -.049467 | .0408266 | .456 | -.150115 | .051182 |
|  |  | TplP | -.023624 | .0365164 | .795 | -.113647 | .066398 |
|  | TplP | EplP | -.025842 | .0365164 | .761 | -.115865 | .064181 |
|  |  | MplP | .023624 | .0365164 | .795 | -.066398 | .113647 |

*Note: Based on observed means, the error term is Mean Square (Error) = .008.*
